# Comparative study of subacute combined degeneration of the spinal cord due to nitrous oxide abuse and vitamin B12 deficiency

**DOI:** 10.3389/fimmu.2025.1567541

**Published:** 2025-04-28

**Authors:** Sedine Marie Desiree Nina Agbo, Xiaohui Duan, Li Wang, Mingrui Dong, Qian Wang, Xiaojing Cai, Fang Liu

**Affiliations:** ^1^ Department of Neurology, The First Hospital of Tsinghua University, Beijing, China; ^2^ Department of Neurology, China-Japan Friendship Hospital, Beijing, China

**Keywords:** nitrous oxide, nitrous oxide abuse, vitamin B12 deficiency, subacute combined degeneration of the spinal cord, peripheral nerve damage, neuroinflammation

## Abstract

**Objective:**

The aim of this study was to compare the clinical presentations, nerve conduction studies, neuroimaging findings of subacute combined degeneration (SCD) caused by N_2_O abuse and primary vitamin B12 deficiency. The goal is to improve diagnostic accuracy, tailored therapeutic interventions, and ultimately enhancing patient outcomes in cases of SCD caused by N_2_O abuse.

**Methods:**

This study was a retrospective case-control study which enrolled 23 patients diagnosed with N_2_O-induced subacute combined degeneration (N_2_O-SCD) and 20 patients with vitamin B12 deficiency-induced subacute combined degeneration (Vit B12-SCD) between 2015 and 2023. Clinical manifestations, physical examinations, laboratory tests, nerve conduction studies, and spinal cord MRI imaging results were collected. Additionally, age-matched healthy control groups were also included for comparative electrophysiological analysis, consisting of 23 young individuals and 21 elderly individuals corresponding to the N_2_O-SCD and Vit B12-SCD groups, respectively.

**Results:**

The study found that compared to Vit B12-SCD, N_2_O-SCD patients exhibited more severe and extensive neurological damage. Both N_2_O-SCD and Vit B12- SCD patients may present with numbness or abnormal sensations, limb weakness, difficulty walking and inability to walk, but these are more severe and widespread in N_2_O-SCD patients. N_2_O-SCD patients showed significant decreases in limb strength, with common walking difficulties and paralysis. Additionally, N_2_O abuse patients more frequently exhibited psychiatric symptoms, especially memory loss, hallucinations and confusion. Both Vit B12-SCD and N_2_O-SCD can cause peripheral nerve demyelination and axonal damage, but it is more severe in the N_2_O-SCD group, with more damage in the lower limbs than in the upper limbs. The extensive nature of axonal damage also indicated a poor prognosis. The degree of spinal cord damage in the N_2_O-SCD group was more severe and affected longer segments. These results suggest that in addition to affecting vitamin B12, N_2_O also causes neurological damage through other mechanisms.

**Conclusion:**

In summary, N_2_O-SCD leads to more severe clinical symptoms, peripheral nerve damage, and spinal cord injury than Vit B12-SCD. These differences guide the clinical treatment of N_2_O-SCD, requiring not only vitamin B12 supplementation but also an addition in neuroprotective treatments.

## 1 Introduction

Subacute combined degeneration (SCD) of the spinal cord is a severe neurological disorder characterized by the degeneration of the dorsal and lateral columns of the spinal cord, leading to a range of motor and sensory dysfunctions. Traditionally, this condition is primarily associated with vitamin B12 deficiency, which impairs myelin synthesis, resulting in demyelination and subsequent neurological deficits (1, 2). However, in recent years, there has been an alarming increase in cases of SCD attributed to the abuse of nitrous oxide (N_2_O), a recreational drug commonly known as “laughing gas” (3, 4). The toxicity related to N_2_O abuse is associated with functional vitamin B12 deficiency, primarily through the inactivation of methionine synthase, a vitamin B12-dependent enzyme crucial for DNA synthesis and myelin production (5, 6). Consequently, N_2_O abuse can precipitate neurological symptoms similar to those seen in classical vitamin B12 deficiency (7, 8).

Clinical observations suggest that SCD resulting from N_2_O abuse may present with more severe and rapid-onset symptoms compared to SCD solely due to vitamin B12 deficiency(9). These symptoms include profound motor and sensory dysfunction, severe paralysis, with incidence of cognitive and psychiatric disorders (10–12). Currently, most N_2_O users are aware of N_2_O poisoning symptoms and hazards, and experienced users deliberately take methylcobalamin supplements to replenish vitamin B12, allowing them to continue extensive N_2_O use. However, this practice has not yielded the desired results; it may delay disease progression without stopping SCD and other neurological or psychiatric damage (13). Paralysis, cognitive, and psychiatric disorders still occur. These findings suggest that besides causing vitamin B12 deficiency, N_2_O abuse may have other potential unknown neurotoxic mechanisms leading to spinal cord neuropathy. Relevant research has also explored the effects of N_2_O on various receptors and channels, including non-competitive inhibition of N-methyl-D-aspartate (NMDA) and non-NMDA glutamate receptors, nicotinic acetylcholine (nACh) receptors, gamma-aminobutyric acid (GABA) receptors, low voltage-activated (T-type) calcium channels, and two-pore domain potassium channels (TREK-1) (14, 15).

This study aims to conduct a comprehensive comparative analysis of SCD caused by N_2_O abuse and SCD due to vitamin B12 deficiency. By examining clinical presentations, neuroimaging findings, biochemical markers, and treatment outcomes, we seek to elucidate the distinct and overlapping features of these two etiologies. Understanding these differences is crucial for improving diagnostic accuracy, tailoring therapeutic interventions, and ultimately enhancing patient outcomes in cases of SCD caused by N_2_O abuse.

## 2 Materials and methods

### 2.1 Subjects

This study enrolled 23 patients diagnosed with N_2_O-induced subacute combined degeneration (N_2_O-SCD) and 20 patients with vitamin B12 deficiency-induced subacute combined degeneration (Vit B12-SCD) between 2015 and 2023. Age-matched healthy control groups were also included for comparative electrophysiological analysis, consisting of 23 young individuals and 21 elderly individuals corresponding to the N_2_O-SCD and Vit B12-SCD groups, respectively. Patients exhibiting polyneuropathy symptoms from other causes or psychiatric disorders were excluded, including conditions such as carpal tunnel syndrome, hyperkalemia, hypokalemia, diabetes, alcohol abuse, uremia, copper or other metal deficiencies, autoimmune diseases, and HIV infection.

This study was approved by the Ethics Committee of the First Hospital of Tsinghua University. Informed consent was obtained from all participants or his/her parents/legal representatives (for participant under 18years old).

### 2.2 Clinical data collection

#### 2.2.1 Clinical evaluation and laboratory assessment

All patients received a comprehensive clinical evaluation, which included a detailed medical history assessment and a physical examination. Laboratory tests were conducted for all patients, encompassing vitamin B12, folate, homocysteine, hemoglobin, and mean corpuscular volume (MCV) tests. Some patients had received vitamin B12 supplementation before examination, so comparisons were made between those who had and had not received treatment prior to laboratory assessment.

#### 2.2.2 Nerve conduction studies (NCS)

Complete motor and sensory NCS were performed using the standard technique. To evaluate the extent of spinal cord and peripheral nerve damage, the patients’ neuroelectrophysiological results were collected. The motor nerve conduction studies included measurements of distal motor latency (DML), motor conduction velocity (MCV), and compound muscle action potential (CMAP) amplitude in the median, ulnar, common peroneal, and tibial nerves. The sensory nerve conduction studies included measurements of sensory conduction velocity (SCV) and sensory nerve action potential (SNAP) amplitude in the median, ulnar, superficial peroneal, and tibial nerves.

CMAP and DML were recorded at the abductor pollicis brevis, abductor digiti minimi, extensor digitorum brevis, and abductor hallucis muscles. SNAP were recorded from the thumb, little finger, Dorsum of the foot and the medial malleolus. Additionally, somatosensory evoked potentials (SSEP) such as N9, N20, N21, P38 were used to detect lesions in the somatosensory conduction pathways. Generally, nerve conduction velocity may slightly slow with age, and waveform morphology may change. Due to significant age differences between the Vit B12-SCD and N_2_O-SCD groups, NCS were compared with age-matched healthy control groups to ensure accuracy and reliability of the results.

#### 2.2.3 Spinal cord magnetic resonance imaging (MRI)

Spinal cord MRI was used to compare spinal cord injuries between the two groups, recording the location, segment, and length of spinal cord damage. This information is typically obtained through MRI T2-weighted images (T2WI) of the spinal cord, identifying the specific site (C: cervical, T: thoracic) and spinal cord segments involved. The length of the injury is estimated by counting the number of affected spinal levels. For example, if the injury extends from C2 to C3, it is considered to involve two spinal segments.

### 2.3 Statistic

All data were statistically analyzed using IBM SPSS (version 29.0) software. Before analysis, the distribution of each group’s data was checked for normality and homogeneity of variance. Clinical manifestations and physical examination results were assessed using chi-square tests, presented as percentages (25%, 50%, 75%). When the expected frequency in any cell was less than 5, Fisher’s exact test was used instead. Laboratory test results, spinal cord lesion lengths, and neuroelectrophysiological results were analyzed as follows: normally distributed continuous variables were analyzed using t-tests, with results presented as mean ± standard deviation; non-normally distributed continuous variables were analyzed using the Mann-Whitney U test, with results presented as median (interquartile range). Statistical graphs were generated using Python. The significance level for the entire study was set at *P <*0.05 (two-tailed test).

## 3 Results

### 3.1 Demographic characteristics of patients

The Vit B12-SCD group enrolled 20 patients (12 males, 8 females) with an average age of 66.2 ± 13.1 years. The disease duration ranged from 1 week to 9 years, with a median duration of 38 weeks and interquartile ranges of 6 to 72 weeks. The N_2_O-SCD group enrolled 23 patients (12 males, 11 females) with an average age of 20.9 ± 3.1 years. The disease duration ranged from 2 weeks to 3 years, with a median duration of 8 weeks and interquartile ranges of 4 to 24 weeks. There was no statistical difference in gender and disease duration between the Vit B12-SCD and N_2_O-SCD groups, but there was a significant age difference (*P <*0.001). Given the differences in electrophysiological reference values due to age, age-matched healthy control groups were enrolled: 21 elderly healthy controls (11 males, 10 females) with an average age of 64.4 ± 13.4 years, and 23 young healthy controls (12 males, 11 females) with an average age of 21.1 ± 3.3 years. There were no statistical differences in gender and age between the Vit B12-SCD group and the elderly control group or between the N_2_O-SCD group and the young control group.

### 3.2 Comparison of clinical symptoms between the Vit B12-SCD and N_2_O-SCD groups

Compared to the Vit B12-SCD group, the N_2_O-SCD group showed a higher incidence of symptoms including confusion, hallucinations, memory loss, and emotional instability (**Table 1**). Additionally, the N_2_O-SCD group had significantly higher rates of limb weakness, difficulty walking, and inability to walk (P< 0.05). In the Vit B12-SCD group, 45% of patients experienced limb weakness, 35% had difficulty walking, and 5% could not walk. Conversely, in the N_2_O-SCD group, 91.3% experienced limb weakness, 73.9% had difficulty walking, and 34.8% could not walk.

**Table 1:** Comparison of clinical symptoms between the Vit B12-SCD and N_2_O-SCD groups.

Symptoms	Vit B12-SCD (N=20)	N_2_O-SCD (N=23)	Pearson Chi- square value	P (Fisher’s test)
Confusion	0 (0%)	6 (26.1%)	6.063	0.023^*^
Hallucination	0 (0%)	7 (30.4%)	7.271	0.010^*^
Delusion	0 (0%)	3 (13%)	2.804	0.236
Abnormal behavior	0 (0%)	4 (17.4%)	3.835	0.111
Memory loss	2 (10%)	11 (47.8%)	7.257	0.009^**^
Emotional instability	1 (5%)	8 (34.8%)	5.734	0.024^*^
Dysarthria	0 (0%)	5 (21.7%)	4.920	0.051
Numbness or paresthesias	17 (85%)	23 (100%)	3.709	0.092
Weakness	9 (45%)	21 (91.3%)	10.874	0.002^**^
Falls	3 (15%)	7 (30.4%)	1.428	0.294
Difficulty walking	7 (35%)	17 (73.9%)	6.568	0.015^*^
Inability to walk	1 (5%)	8 (34.8%)	5.734	0.024^*^
Unsteady gait	14 (70%)	21 (91.3%)	3.206	0.118
Dysuria	5 (25%)	7 (30.4%)	0.157	0.745

*indicates *P*<0.05, **indicates *P*<0.01.

### 3.3 Comparison of clinical signs between the Vit B12-SCD and N_2_O-SCD groups

There were significant differences in muscle tone, muscle strength, sensory abnormalities, and other neurological dysfunctions between the two groups (**Table 2**). In terms of muscle tone, 15% of patients in the Vit B12-SCD group exhibited increased muscle tone, compared to 13% in the N_2_O-SCD group. Notably, 34.8% of patients in the N_2_O-SCD group showed decreased muscle tone, a phenomenon not observed in the Vit B12-SCD group. Sensory disturbances also showed notable differences. Approximately 82.6% of patients in the N_2_O-SCD group reported sensory abnormalities, significantly higher than the 45% in the Vit B12-SCD group.

**Table 2:** Comparison of clinical signs between the Vit B12-SCD and N_2_O-SCD groups.

Clinical sign		Vit B12-SCD (N=20)	N_2_O-SCD (N=23)	Pearson Chi- square value	P (Fisher’s test)
Muscle tone	↓	0 (0%)	8 (34.8%)	8.695	0.007^**^
	↑	3 (15%)	3 (13%)		
Muscle strength < grade 3	+	0 (0%)	13 (56.5%)	16.203	<0.001^***^
Upper limb reflex	+	4 (20%)	7 (30.4%)	2.539	0.694
	++	11 (55%)	8 (34.8%)		
	+++	1 (5%)	2 (8.7%)		
	++++	0 (0%)	1 (4.3%)		
Lower limb reflex	+	10 (50%)	7 (30.4%)	7.212	0.111
	++	5 (25%)	2 (8.7%)		
	+++	0 (0%)	3 (13%)		
	++++	1 (5%)	1 (4.3%)		
Babinski sign	+	10 (50%)	7 (30.4%)	1.713	0.160
Hypoesthesia	+	9 (45%)	19 (82.6%)	6.661	0.011^*^
Vibration sensation	↓	15 (75%)	15 (65.2%)	0.485	0.360
Lhermitte sign	+	2 (10%)	11 (47.8%)	7.257	0.008^**^
Romberg sign	+	12(60%)	15 (65.2%)	0.380	0.908
	non cooperative	3 (15%)	4 (17.4%)		
Ataxia	+	8 (40%)	6 (26. 1%)	1.656	0.515
	non cooperative	0 (0%)	1 (4.3%)		

*Indicates *P*<0.05, **indicates *P*<0.01, ***indicates *P*<0.001.

**Figure 1:**
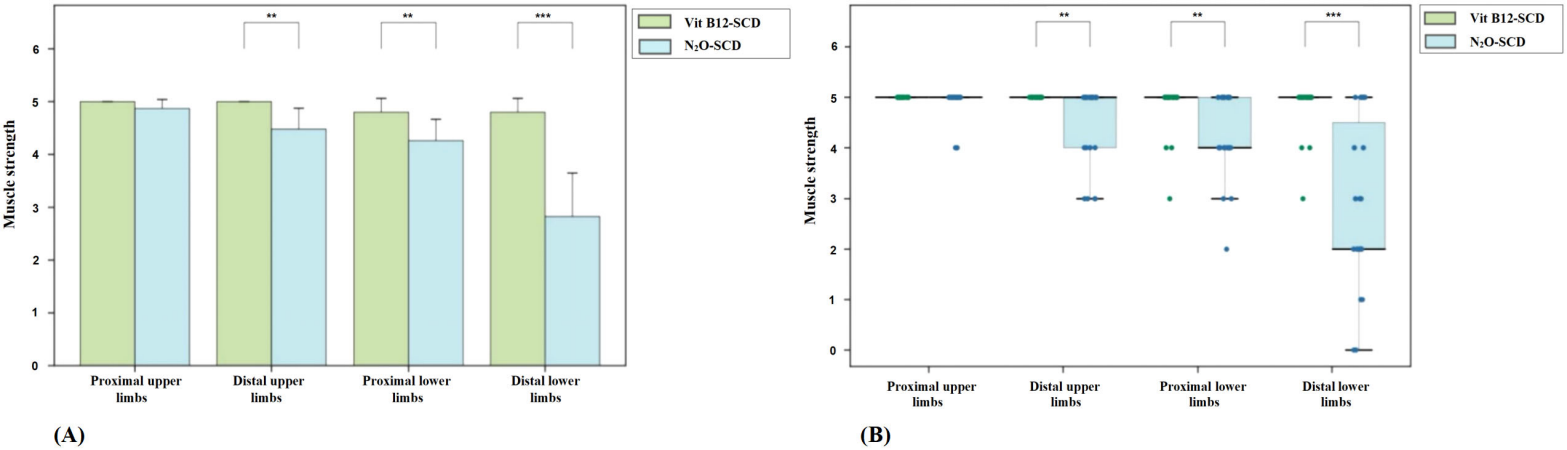
Distribution of muscle strength across all four limbs in Vit B12-SCD and N_2_O-SCD. **(A)** shows the comparison of muscle strength in all four limbs between Vit B12-SCD and N_2_O-SCD; **(B)** shows the distribution of muscle strength across all four limbs between Vit B12-SCD and N_2_O-SCD. Vertical axis: muscle strength. Horizontal axis: Examined nerves. **indicates *P* <0.01, ***indicates *P* <0.001.

Regarding muscle strength, patients in the Vit B12-SCD group generally maintained muscle strength between grades 3 to 5, while 56.5% of patients in the N_2_O-SCD group had lower limb muscle strength below grade 3 (see **Figure 1**). This difference was further highlighted by comparisons of proximal and distal muscle strength in the limbs. Notably, although the average muscle strength score for the distal upper limbs was 5.0 in both groups, statistical analysis revealed a significant difference (*P <*0.002). Similarly, comparisons of proximal and distal muscle strength in the lower limbs showed significant differences, particularly in the distal lower limbs, where the average score in the N_2_O-SCD group was 2.0 (range 2.0 to 4.5), significantly lower than in the Vit B12-SCD group (*P <*0.001).

Furthermore, 47.8% of patients in the N_2_O-SCD group exhibited Lhermitte’s sign, a proportion significantly higher than in the Vit B12-SCD group, possibly indicating more severe spinal cord damage caused by N_2_O-SCD.

### 3.4 Laboratory findings

Among patients who had not received vitamin B12 supplementation before laboratory examination, the Vit B12-SCD group had 16, 14, and 14 patients, while the N_2_O-SCD group had 12, 12, and 15 patients who completed vitamin B12, folate, and homocysteine tests, respectively. Additionally, among those who had received or taken doses of vitamin B12 before the laboratory assessment, the Vit B12-SCD group had 2, 2, and 3 patients, and the N_2_O-SCD group had 9, 9, and 7 patients who completed these tests, respectively. All patients in the N_2_O-SCD group completed hemoglobin and MCV tests, while only 19 patients in the Vit B12-SCD group completed these tests.

There were significant differences in vitamin B12 levels between the Vit B12-SCD and N_2_O-SCD groups among patients who had not received treatment prior to admission (see **Table 3**). The median vitamin B12 level in the Vit B12-SCD group was 75.1 pg/mL (range 60.9 to 176.1 pg/mL), significantly lower than the median level in the N_2_O-SCD group, which was 162.0 pg/mL (range 114.0 to 413.0 pg/mL), with a P value of 0.030. There were no significant differences in folate and homocysteine levels between the Vit B12-SCD and N_2_O-SCD groups. Among patients who received vitamin B12 treatment (see **Table 4**), levels of vitamin B12, folate, and homocysteine did not show statistically significant differences. There was also no differences found between the two groups in hemoglobin and MCV levels.

**Table 3:** Laboratory examination prior to vitamin B12 supplementation.

Laboratory examination	Vit B12-SCD	N_2_O-SCD	P
Vitamin B12 (133–675 pmol/L)	75.1 (60.9 to 176.1)	162.0 (114.0 to 413.0)	0.030^*^
Folate (7.0-45.1 nmol/L)	35.9 (15.7 to 45.4)	17.96 (14.0 to 29.8)	0.145
Homocysteine (≤15μmol/L)	27.1 (13.8 to 120.0)	30.7 (17.1 to 60.9)	0.813

*Indicates *P*<0.05.

**Table 4:** Comparison of SSEP between N_2_O-SCD group and Vit B12-SCD group.

SSEP	Vit B12-SCD(ms)	N_2_O-SCD (ms)	P
Cortical Potential(N20)	N= 8	N= 8	
	20.2 (18.6 to 22.5)	20.4 (19.5 to 21.8)	0.840
Subcortical Potential(N9)	N= 8	N= 8	
	9.5 (8.7 to 9.8)	9.4 (9.0 to 10.3)	0.545
Cortical Potential (P38)	N= 12	N= 8	
	45.9 (43.1 to 47.0)	45.6 (20.7 to 49.2)	0.976
Subcortical Potential(N21)	N= 12	N= 8	
	10.0 (0.0 to 21.30)	0.0(0.0 to 0.0)	0.028^*^

*Indicates *P <*0.05.

### 3.5 Electrophysiological characteristics

#### 3.5.1 Comparison between N_2_O-SCD and Vit B12-SCD groups

##### 3.5.1.1 Comparison of motor nerve conduction

**Figure 2:**
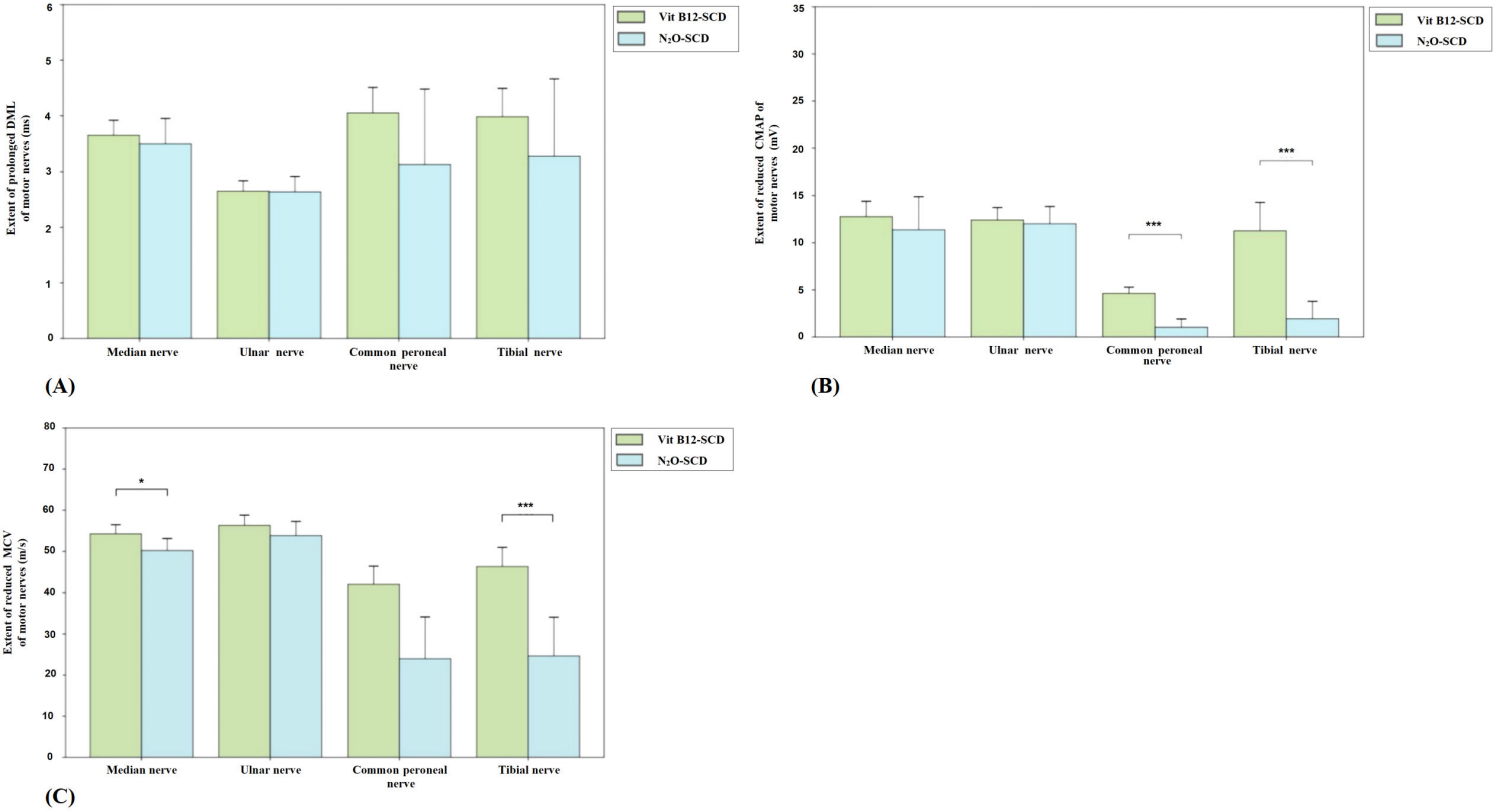
Comparison of motor nerve conduction between N_2_O-SCD group and Vit B12-SCD group. **(A)**: Distal Motor Latency (DML, ms); **(B)**: Compound Muscle Action Potential (CMAP, mV); and **(C)**: Motor Conduction Velocity (MCV, m/s). Vertical axis: **(A)** extent of prolonged DML, **(B)** extent of reduced CMAP, **(C)** extent of reduced MCV. Horizontal axis: examined nerves. *Indicates *P <*0.05, ***indicates *P <*0.001.

**Figure 2** presents the comparison of DML, CMAP, and MCV measurements for the median, ulnar, common peroneal, and tibial nerves between the N_2_O-SCD and Vit B12-SCD groups. In the Vit B12-SCD group, motor nerve conduction was completed for 19 out of 20 patients for the median and tibial nerves, 12 patients for the common peroneal nerve, and 18 patients for the ulnar nerve. In the N_2_O-SCD group, motor nerve conduction was completed for 20 patients for the common peroneal nerve and for 23 patients for the median, ulnar, and tibial nerves. No significant differences were found between the N_2_O-SCD and Vit B12-SCD groups in the DML, CMAP, and MCV of the ulnar nerve. However, a significant difference was observed in the median nerve conduction velocity between the two groups (*P <*0.05), with values of 54.3 ± 4.4 m/s in the Vit B12-SCD group and 50.2 ± 5.83 m/s in the N_2_O-SCD group. Additionally, the N_2_O-SCD group exhibited significantly reduced CMAP and MCV in the common peroneal and tibial nerves compared to the Vit B12-SCD group (*P <*0.001).

##### 3.5.1.2 Comparison of sensory nerve conduction

In the Vit B12-SCD group, among the 20 patients, 19 completed the SCV and SNAP measurements for the median and tibial nerves, 18 completed these measurements for the ulnar nerve, and 12 completed them for the superficial peroneal nerve. In the N_2_O-SCD group, among the 23 patients, 20 completed SCV and SNAP measurements for the superficial peroneal nerve, 22 completed these measurements for the tibial nerve, and all 23 patients completed these measurements for the median and ulnar nerves.

There were no significant differences in SCV for the median and ulnar nerves in the upper limbs between the two groups. However, significant differences were observed in sensory nerve action potential (SNAP) amplitudes. The median nerve SNAP amplitude was significantly lower in the Vit B12-SCD group compared to the N_2_O-SCD group (9.8 µV vs. 18.5 µV, *P <*0.01). Similarly, the ulnar nerve SNAP amplitude was significantly lower in the Vit B12-SCD group (5.1 µV vs. 10.4 µV, *P <*0.001).

**Figure 3:**
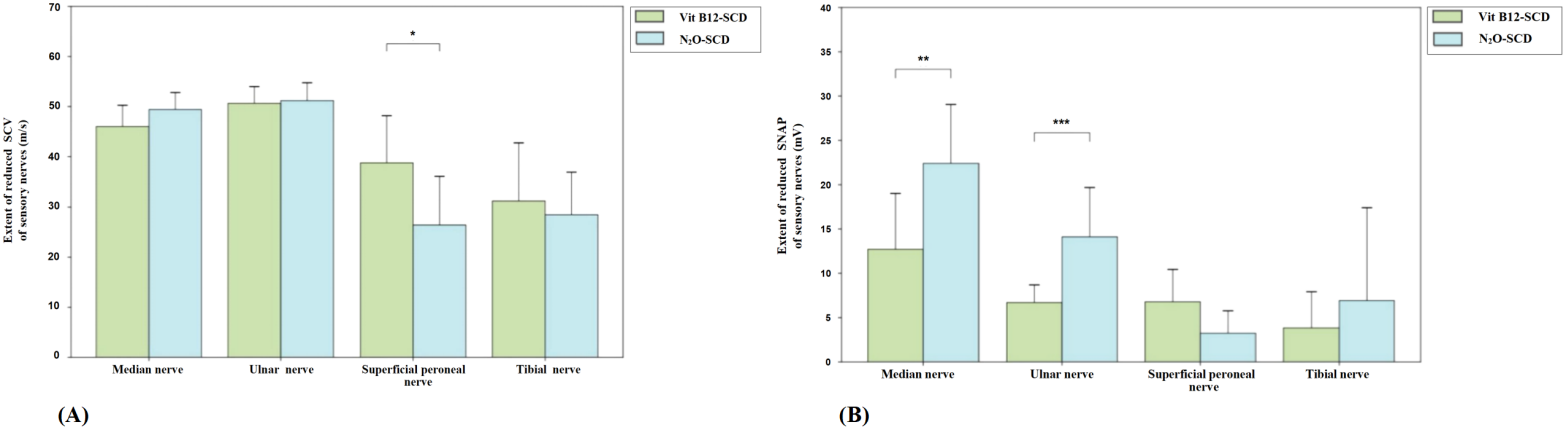
Comparison of sensory nerve conduction between N_2_O-SCD group and Vit B12-SCD group. **(A)**: Sensory Conduction Velocity (SCV, m/s); **(B)**: Sensory Nerve Action Potential (SNAP, mV). Vertical axis: **(A)** extent of reduced SCV, **(B)** extent of reduced SNAP. Horizontal axis: examined nerves. *Indicates *P <*0.05, **indicates *P <*0.01, ***indicates *P <*0.001.

In contrast, for the superficial peroneal nerve in the lower limbs, the N_2_O-SCD group showed significantly slower SCV (median 33.05 m/s) compared to the Vit B12-SCD group (43.6 m/s, *P <*0.05). However, the decrease in SNAP amplitude for the superficial peroneal nerve did not reach statistical significance (see **Figure 3**).

#### 3.5.2 Comparison of Vit B12-SCD group with elderly control group

##### 3.5.2.1 Comparison of motor nerve conduction

**Figure 4:**
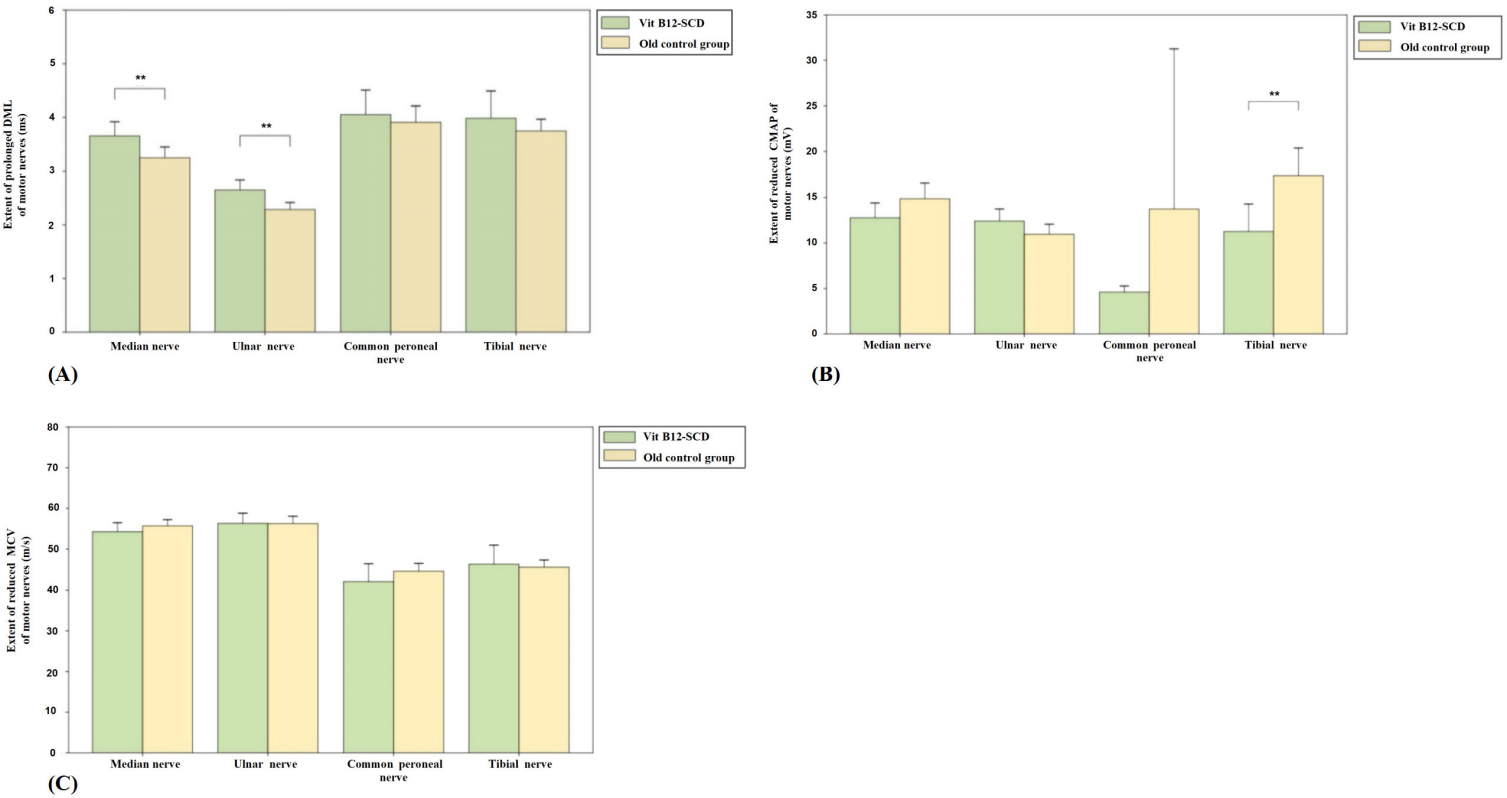
Comparison of motor nerve conduction between Vit B12-SCD and old control group. **(A)**: Distal Motor Latency (DML, ms); **(B)**: Compound Muscle Action Potential (CMAP, mV); and **(C)**: Motor Conduction Velocity (MCV, m/s). Vertical axis: **(A)** extent of prolonged DML, **(B)** extent of reduced CMAP, **(C)** extent of reduced MCV. Horizontal axis: examined nerves. **Indicates *P <*0.01.

In the Vit B12-SCD group, the DML of the median and ulnar nerves was significantly prolonged compared to the elderly control group. The median nerve DML increased from 3.1 ms to 3.7 ms (*P <*0.01) and the ulnar nerve DML increased from 2.3 ± 0.3 ms to 2.7 ± 0.4 ms (*P <*0.01) in the elderly control group and Vit B12-SCD group respectively. Although the CMAP of the median, common peroneal, and tibial nerves decreased in the Vit B12-SCD group compared to the control group, only the reduction in the tibial nerve CMAP was statistically significant (from 17.4 ± 6.0 mV to 11.3 ± 6.0 mV, *P <*0.01). There was no significant difference in the MCV of all tested nerves between the elderly control group and the Vit B12-SCD group (see **Figure 4**).

##### 3.5.2.2 Comparison of sensory nerve conduction

**Figure 5:**
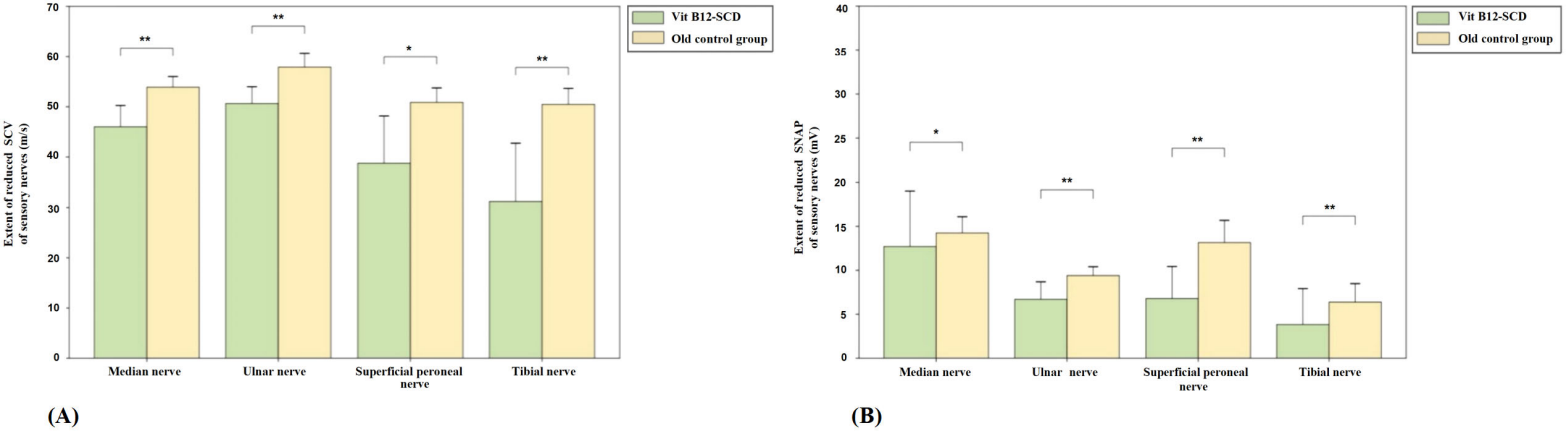
Comparison of sensory nerve conduction between Vit B12-SCD and old control group. **(A)**: Sensory Conduction Velocity (SCV, m/s); **(B)**: Sensory Nerve Action Potential (SNAP, mV). Vertical axis: **(A)** extent of reduced SCV, **(B)** extent of reduced SNAP. Horizontal axis: examined nerves. *Indicates *P <*0.05, **indicates *P <*0.01.

**Figure 5** shows the comparison of sensory nerve conduction between the Vit B12-SCD group and the elderly control group. There were significant differences in SCV for the median (51.9 ± 4.2 m/s vs. 46.0 ± 8.5 m/s), ulnar (median 57.7 m/s vs. 51.9 m/s), superficial peroneal (50.8 ± 5.8 m/s vs. 38.8 ± 18.8 m/s), and tibial nerves (50.5 ± 6.4 m/s vs. 31.2 ± 23.0 m/s), with the Vit B12-SCD group showing significantly slower SCV (*P <*0.01). Additionally, there were significant differences in SNAP for the median (12.7 ± 12.6 μV vs. 14.2 ± 3.7 μV), ulnar (6.7 ± 4.0 μV vs. 9.4 ± 2.0 μV), superficial peroneal (6.8 ± 7.3 μV vs. 13.2 ± 5.1 μV), and tibial nerves (3.8 ± 8.2 μV vs. 6.4 ± 4.2 μV), Vit B12-SCD and elderly control group respectively. Both group’s SCV in the ulnar nerve were within the normal range, despite statistical differences being observed.

#### 3.5.3 Comparison of N_2_O-SCD group with young control group

##### 3.5.3.1 Comparison of motor nerve conduction

**Figure 6:**
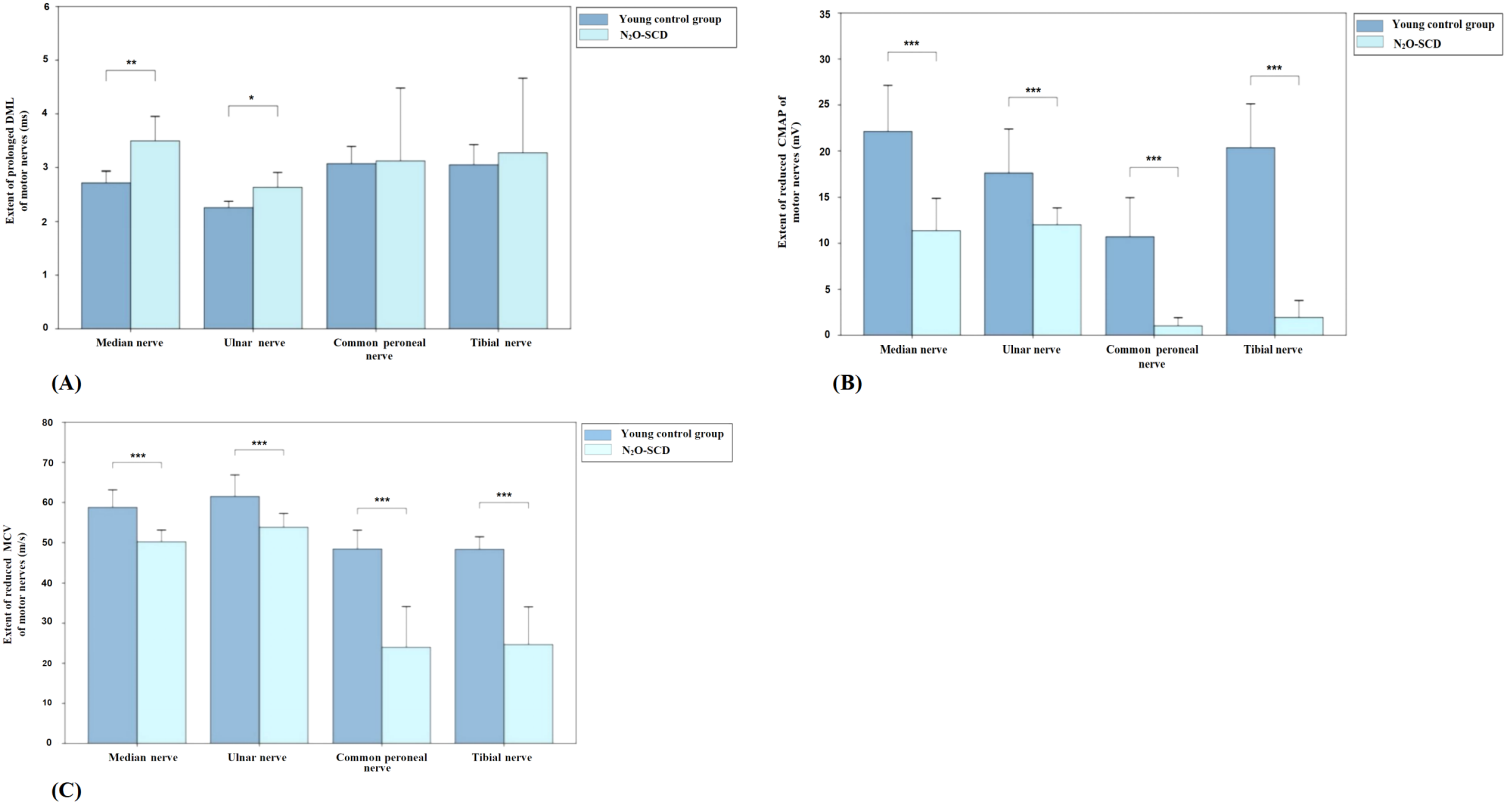
Comparison of motor nerve conduction between N_2_O-SCD group and young control group. **(A)**: Distal Motor Latency (DML, ms); **(B)**: Compound Muscle Action Potential (CMAP, mV); and **(C)**: Motor Conduction Velocity (MCV, m/s). Vertical axis: **(A)** extent of prolonged DML, **(B)** extent of reduced CMAP, **(C)** extent of reduced MCV. Horizontal axis: examined nerves. *Indicates *P <*0.05, **indicates *P <*0.01, ***indicates *P <*0.001.

**Figure 6** shows the comparison of motor nerve conduction between the N_2_O-SCD group and the young control group. The median nerve DML was significantly prolonged (2.7 ms vs. 3.4 ms, *P <*0.01) and MCV was significantly reduced (50.2 m/s vs. 60.5 m/s, *P <*0.001) in the N_2_O-SCD group. Additionally, the median nerve CMAP was significantly reduced (11.4 ± 7.0 mV vs. 20.4 ± 4.8 mV, *P <*0.001). The ulnar nerve showed a similar trend, with significantly prolonged DML (2.6 ± 0.6 ms vs. 2.2 ± 0.2 ms, *P <*0.05), reduced CMAP (12.4 mV vs. 15.4 mV, *P <*0.001), and slower MCV (53.8 ± 6.9 m/s vs. 63.3 ± 4.6 m/s, *P <*0.001) in the N_2_O-SCD group.

The differences in the lower limbs’ common peroneal nerve and tibial nerve are more pronounced. In the N_2_O-SCD group, the common peroneal nerve’s CMAP was significantly reduced compared to the young control group (0.3 mV vs. 9.2 mV, *P <*0.001), and the nerve conduction velocity (MCV) was significantly slower (36.7 m/s vs. 49.7 m/s, *P <*0.001). Similarly, the tibial nerve in the N_2_O-SCD group showed a significantly lower CMAP compared to the young control group (0.1 mV vs. 18.5 mV, *P <*0.001), and the MCV was significantly slower (34.3 m/s vs. 49.5 m/s, *P <*0.001).

##### 3.5.3.2 Comparison of sensory nerve conduction between N_2_O-SCD group and young control group

**Figure 7:**
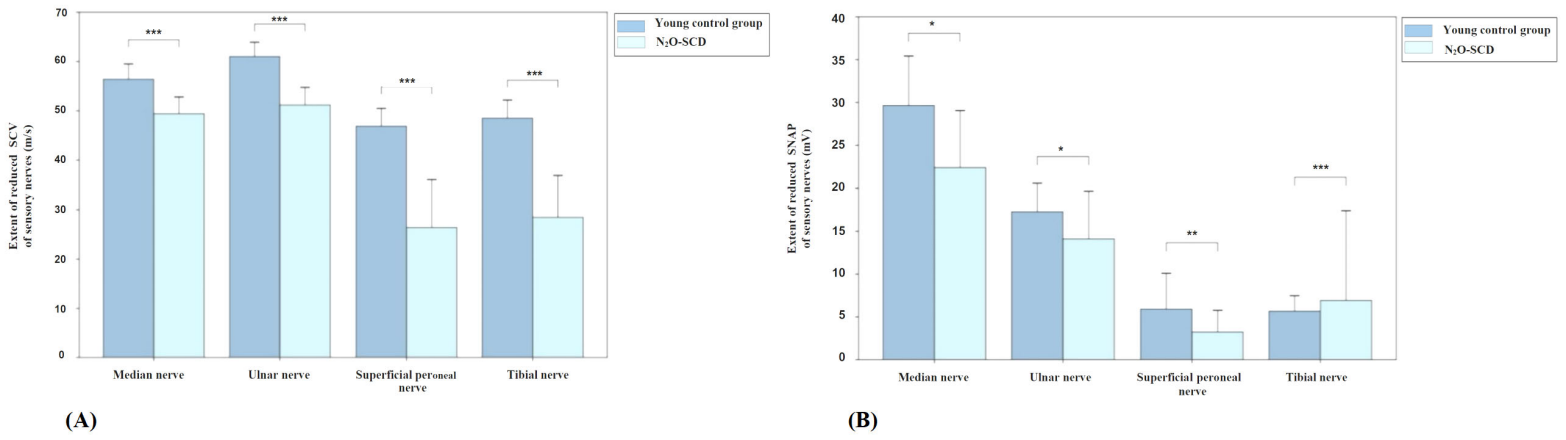
Comparison of sensory nerve conduction between N_2_O-SCD and young control group. **(A)**: Sensory Conduction Velocity (SCV, m/s); **(B)**: Sensory Nerve Action Potential (SNAP, mV). Vertical axis: **(A)** extent of reduced SCV, **(B)** extent of reduced SNAP. Horizontal axis: examined nerves. *indicates *P <*0.05, **indicates *P <*0.01, ***indicates *P <*0.001.

**Figure 7** shows the comparison results of sensory nerve conduction between the N_2_O-SCD group and the young control group. SCV comparison reveals significant differences in the median nerve (49.4 ± 6.8 m/s vs. 56.8 ± 6.4 m/s), ulnar nerve (51.2 ± 7.1 m/s vs. 61.0 ± 5.7 m/s), superficial peroneal nerve (31.2 m/s vs. 44.8 m/s), and tibial nerve (36.0 m/s vs. 46.4 m/s), in the N_2_O-SCD and young control group respectively. SCV in the N_2_O-SCD group was significantly slower than in the young control group (*P <*0.001). The sensory nerve action potential (SNAP) comparison shows that the SNAPs in the N_2_O-SCD group were significantly lower than those in the young control group for the median nerve (18.5 uV vs. 28.8 uV, *P <*0.05), ulnar nerve (10.4 uV vs. 17.8 uV, *P <*0.05), superficial peroneal nerve (1.1 uV vs. 2.6 uV, *P <*0.01), and tibial nerve (2.4 uV vs. 4.9 uV, *P <*0.001).

### 3.6 Somatosensory evoked potentials

When comparing the SSEP between the two groups, we found that only the subcortical potential (N21) showed a statistically significant difference (*P <*0.05). In the N_2_O-SCD group, no latency was elicited in any patients (see **Table 4**).

### 3.7 Imaging study analysis

**Figure 8:**
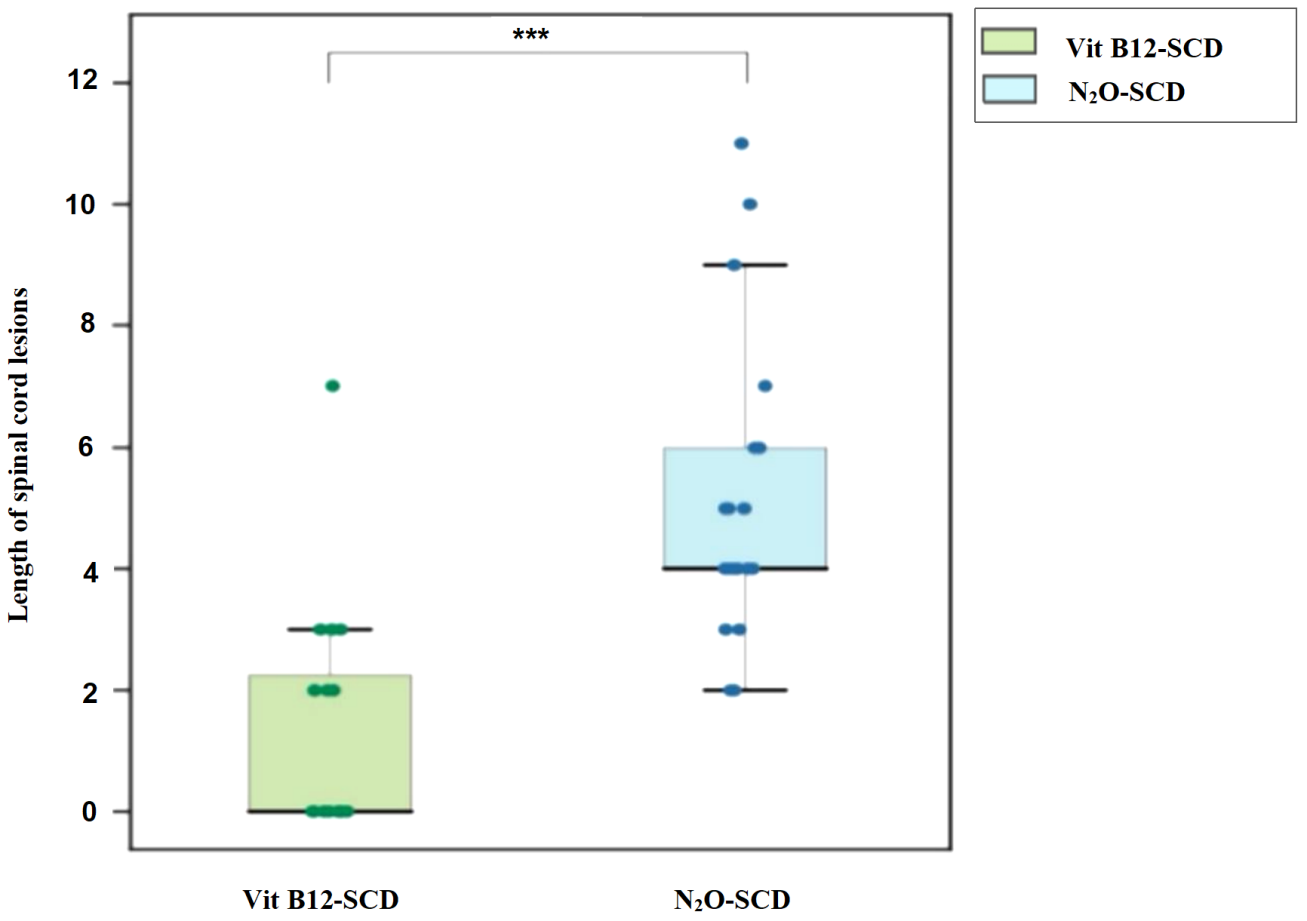
Distribution of spinal cord lesions. Vertical axis: Length of spinal cord lesions. ***indicates *P* <0.001.

**Figure 9:**
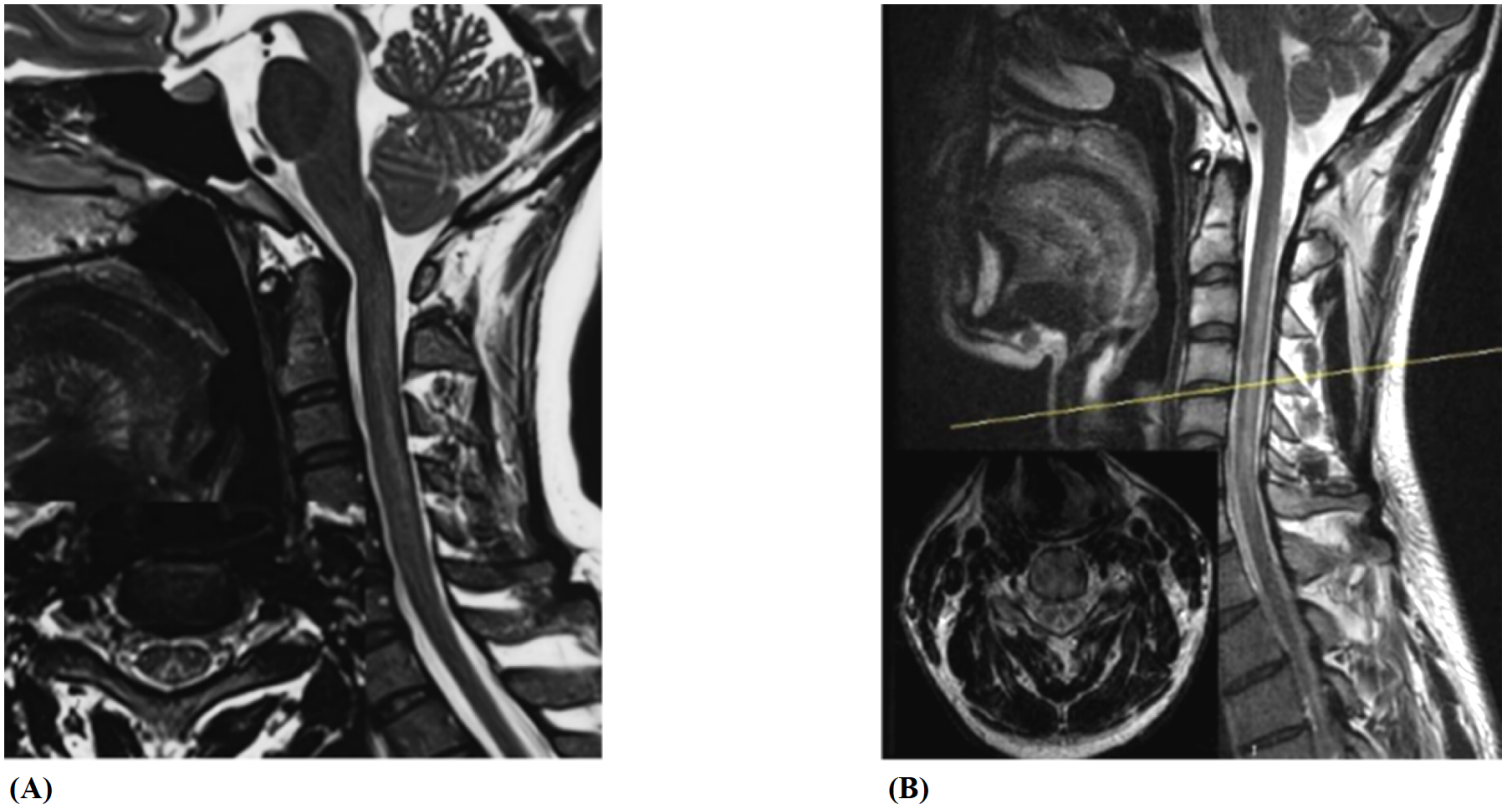
Spinal cord lesions. **(A, B)** show spinal cord lesions in a patient with primary vitamin B12 deficiency and in a patient with nitrous oxide abuse, respectively.

Both the N_2_O-SCD group (23 cases) and the Vit B12-SCD group (20 cases) completed spinal cord MRI. The analysis of damage locations in the cervical, thoracic, and cervicothoracic regions showed statistically significant differences only in cervical lesions (*P <*0.001) (see **Table 5**). When comparing the length of spinal cord lesion segments between the two groups, significant differences were found (*P <*0.001) (see **Figure 8**). The average length of spinal cord lesions in the N_2_O-SCD group was 5.1 ± 2.3 segments, whereas in the Vit B12-SCD group, it was 1.3 ± 1.9 segments. **Figure 9** shows a spinal cord lesions in a patient with primary vitamin B12 deficiency and in a patient with nitrous oxide abuse where we can see the differences in length in both types of patients.

**Table 5:** Spinal cord lesion segments.

Spinal Cord Lesion Segments	Vit B12-SCD (N=20)	N_2_O-SCD (N=23)	P
Cervical Segment	5(25%)	23(100%)	< 0.001^***^
Thoracic Segment	4(20%)	5(21.7%)	0.595
Cervical + Thoracic Segment	1(5%)	5(21.7%)	0.192

***Indicates *P*<0.001.

## 4 Discussion

The abuse of N_2_O has become increasingly popular in Western and Asian countries, particularly among adolescents and young adults (16–21). N_2_O is a colorless, sweet-tasting gas with significant psychoactive effects (22). Its abuse can lead to various types of neuropsychiatric damage, including subacute combined degeneration (SCD), peripheral neuropathy, and cognitive and psychiatric disorders. Both primary vitamin B12 deficiency and N_2_O abuse-induced spinal cord involvement can impair vibration sense, position sense, and touch, often accompanied by sensory ataxia (gait instability) and a positive Romberg sign. Peripheral nerve involvement typically manifests as sensory and motor disturbances. Sensory disturbances usually present as symmetrical, distal, glove-and-stocking-like paresthesia. Motor disturbances present as limb weakness and difficulty walking. Pathological signs and deep reflex abnormalities are also common (12, 23). Psychiatric disturbances such as hallucinations, confusion, delusions, mood disorders, memory loss, and autonomic symptoms are less common (7, 22, 24).

### 4.1 N_2_O abuse leads to greater cognitive and psychiatric impairment compared to vitamin B12 deficiency

In this study, compared with Vit B12-SCD group, the N_2_O-SCD group showed higher incidences of confusion, hallucinations, memory loss, emotional instability, limb weakness, and difficulty walking. Memory loss was the most common symptom in N_2_O-induced SCD and less prevalent in Vit B12-SCD group. These findings suggest that N_2_O abuse may trigger more severe psychiatric and neurological symptoms due to its neurotoxic effects, which likely involve both neuroinflammation and disruption of neurotransmission. N_2_O abuse induces inflammatory responses by elevating pro-inflammatory cytokines such as IL-6 and TNF-alpha, which activate microglia and lead to the release of reactive oxygen species (ROS) and nitric oxide (NO), causing oxidative stress and neuronal damage. This neuroinflammation is linked to an increased risk of neurodegenerative diseases (25) and contributes to cognitive decline, particularly in the hippocampus, which is crucial for memory and learning (26, 27). In addition to neuroinflammation, N_2_O also antagonizes NMDA receptors, which play a vital role in synaptic plasticity and memory formation. The inhibition of NMDA receptors in the hippocampus disrupts neurotransmission, further impairing cognitive function (28, 29). This disruption of NMDA receptor function, combined with the inflammatory cytokine response, likely exacerbates neuronal injury and contributes to the higher incidence of psychiatric and neurological symptoms observed in the N_2_O-SCD group, highlighting the heightened neurotoxic potential of N_2_O compared to Vitamin B12 deficiency.

### 4.2 More severe, extensive sensory loss and motor decline in N_2_O abuse compared to vitamin B12 deficiency

Many studies indicate that common clinical symptoms of N_2_O abuse include numbness, limb weakness, and gait instability (9, 30–32). In this study, we also found that the N_2_O patients predominantly experience numbness or paresthesia, limb weakness, and gait instability, pointing to severe neurological impairment. This high prevalence underscores the significant neurotoxic effects of nitrous oxide abuse on peripheral nerves and spinal cord integrity. In contrast, the Vit B12-SCD group exhibits a different pattern of clinical manifestations. Numbness or paresthesia and gait instability are most common, followed by limb weakness (2, 31). This suggests that while both conditions share overlapping symptoms, the severity and distribution of neurological impairments differ, reflecting their distinct pathophysiological mechanisms. Early walking difficulties arise from sensory disturbances but can be compensated by muscle strength. This compensation is expected to be stronger in adolescents. However, our study show that walking difficulties are more severe in young people with N_2_O abuse, indicating that spinal cord and peripheral nerve damage from nitrous oxide is more severe in young individuals compared to the damage caused by vitamin B12 deficiency in older individuals. Furthermore, the severity of muscle weakness especially in the lower limbs can greatly impair mobility and stability. However, the pattern of muscle strength decline differs between the two conditions. N_2_O abuse typically results in more severe muscle strength decline in the lower and distal limbs compared to the upper and proximal limbs (32). This pattern indicates a length-dependent neuropathy, characteristic of toxic peripheral neuropathy. In the N_2_O-SCD group, muscle strength decline is notably more significant than in the Vit B12-SCD group, with a greater proportion of patients experiencing severe lower limb weakness. This suggests that the neurotoxic effects of N_2_O disproportionately affect the longer peripheral nerves, leading to more pronounced distal limb impairment.

Although both groups reported numbness and paresthesia, objective examinations revealed significant differences. The N_2_O-SCD group showed more extensive glove-and-stocking sensory loss, extending to the neck and chest, indicating more severe and widespread peripheral neuropathies. In contrast, the Vit B12-SCD group had less extensive sensory abnormalities, with no involvement of the neck and chest. These findings suggest that neurological damage is more severe in the N_2_O-SCD group compared to the Vit B12-SCD group. Previous studies have shown that in N_2_O abusing patients, sensory abnormalities and sensory ataxia often persist long-term after treatment and may not fully recover (11, 37). The persistence of sensory abnormalities and the presence of sensory ataxia highlight the long-term and potentially irreversible impact of N_2_O abuse on the nervous system. The distribution of sensory reduction also points to a more widespread and severe pattern of neurological impairment in N_2_O-SCD, affecting both peripheral nerves and the spinal cord more profoundly than in Vit B12-SCD.

### 4.3 More severe axonal and myelin dysfunction in N_2_O abuse compared to vitamin B12 deficiency

In neuroelectrophysiological assessments, both N_2_O-SCD and Vit B12-SCD groups exhibited axonal and myelin sheath damage, with distinct differences. The N_2_O-SCD group showed significantly slower conduction velocities in the median and tibial nerves, indicating more severe myelin damage in limb motor nerves, and reduced amplitudes in the common peroneal and tibial nerves, suggesting greater axonal damage in the lower limbs. The Vit B12-SCD group had lower SNAP amplitudes in the median and ulnar nerves, pointing to more severe upper limb sensory axonal damage, likely due to a combination of vitamin B12 deficiency and potential age-related factors. Additionally, the N_2_O-SCD group displayed slower sensory conduction velocity in the superficial peroneal nerve, indicating more extensive myelin sheath damage in the lower limbs. Somatosensory evoked potentials further confirmed impaired conduction in lower limb nerve pathways, consistent with Tani et al.’s (33) findings of more severe motor nerve axonal damage and sensory nerve myelin sheath damage in the N_2_O-SCD group compared to the Vit B12-SCD group. This study also highlighted more severe myelin sheath damage in both upper and lower limb motor nerves in the N_2_O-SCD group. In comparing the Vit B12-SCD group to a control group of healthy elderly individuals, our findings indicate that vitamin B12 deficiency leads to demyelination and axonal damage in both upper and lower limb sensory nerves. This comparison underscores that these neurological impairments are primarily related to vitamin B12 deficiency, though age-related changes might exacerbate the damage. When the N_2_O-SCD group was compared to a young control group, there was evidence of simultaneous motor and sensory nerve involvement in both upper and lower limbs, characterized by axonal degeneration and demyelination, with more severe damage in the lower limbs. This is consistent with existing literature showing that peripheral nerve damage in N_2_O abuse primarily involves axonal damage, followed by mixed-type and myelin sheath damage. These findings suggest that while vitamin B12 deficiency alone can cause significant nerve damage, the addition of N_2_O abuse leads to much more severe axonal degeneration and demyelination. This exacerbated damage in N_2_O-SCD likely results from the combined effects of vitamin B12 deficiency and the neurotoxic properties of N_2_O, which disrupts neural function through mechanisms like NMDA receptor antagonism and ischemic changes (34). Demyelination can trigger axonal damage by disrupting transport and causing degeneration, while axonal degeneration can lead to secondary demyelination due to the loss of axonal support. Inflammatory mediators released during demyelination promote further axonal damage, creating a vicious cycle (35). Demyelinating lesions may have a better prognosis due to the potential for remyelination, especially if N_2_O abuse is halted. However, axonal damage is often permanent, with motor nerve recovery dependent on slower distal reinnervation (35). N_2_O -induced neuropathy tends to cause early demyelination, but axons are particularly vulnerable due to their high metabolic demands and limited repair capacity (36). Even without significant demyelination, axons remain at risk. There is no correlation between nerve electrophysiology and vitamin B12 levels, homocysteine, or hemoglobin (32), but chronic N_2_O abuse, especially over 80g/day, is linked to irreversible nerve damage and poor prognosis (37). Subacute neuropathy from N_2_O abuse develops rapidly, causing severe impairment, though higher motor nerve amplitudes in the common peroneal and tibial nerves may suggest a better prognosis (36).

### 4.4 Greater spinal cord damage and longer lesions in N_2_O abuse compared to vitamin B12 Deficiency

On another note, patients in both groups exhibited Lhermitte’s sign, but its higher incidence in the N_2_O-SCD group suggests more severe spinal cord pathology. MRI confirmed more extensive and severe spinal cord lesions in the N_2_O-SCD group, predominantly affecting the cervical region and extending into the thoracic spine (≥3 vertebral segments), with lesions primarily in the cervical posterior column. In contrast, the Vit B12-SCD group had shorter, cervical-only lesions. Vitamin B12 deficiency-related myelopathy, as described by Scalabrino et al. (38), involves cytokine and growth factor imbalances, leading to myelin swelling and degeneration and elevated TNF-α and reduced IL-6 levels. N_2_O abuse, however, induces neurotoxic effects through mechanisms like p53 upregulation via nNOS activation, mitochondrial damage, and subsequent demyelination (39–41). N_2_O also inhibits spinal cord c-Fos activity, affecting gene expression and inflammatory responses, likely worsening spinal cord damage in N_2_O-SCD compared to Vit B12-SCD (39). Wu et al.’s (42) case study highlights worsening symptoms after initial vitamin B12 treatment, with MRI revealing persistent spinal cord damage despite clinical improvement and normal blood tests, similar to delayed effects seen in central pontine myelinolysis (26). Thus, MRI changes may reflect both current and past myelin damage, edema, or structural alterations, emphasizing the complex and varied nature of spinal cord injury in these conditions.

### 4.5 The effect of N_2_O abuse on vitamin B12 and homocysteine concentrations alongside neurological functionality

N_2_O abuse can lead to inconsistent changes in vitamin B12 levels, especially in individuals who have already received vitamin B12 supplementation (43). In this study, we found that patients who had not received vitamin B12 supplementation before admission had significantly lower vitamin B12 levels in the Vit B12-SCD group compared to the N_2_O-SCD group. However, despite the lower vitamin B12 levels, the clinical manifestations were milder in the Vit B12-SCD group. This suggests that N_2_O abuse not only causes vitamin B12 deficiency but may also impair the activity and function of vitamin B12 and that the severity of clinical symptoms may not be directly linked to measured vitamin B12 levels and may involve others factors. These findings are consistent with Garakani et al.’s (12) conclusions, which noted that serum vitamin B12 levels in N_2_O abusers are often reduced or low-normal, indicating that serum vitamin B12 is not a reliable marker for N_2_O toxicity (23), despite vitamin B12 deficiency being the gold standard for diagnosing Vit B12-SCD (43).

High homocysteine (Hcy) levels are known to increase oxidative stress, generating free radicals that damage myelin and nerve cells, leading to demyelination and functional impairments (6). Briani et al. (2) suggested that elevated Hcy levels, which rise rapidly after N_2_O inhalation, could serve as a marker of recent N_2_O exposure and toxicity. Additionally, Kondo et al. (44) reported that the inhibition of methionine synthase (MS) activity following N_2_O exposure could explain the resulting rise in Hcy levels. In this study, we observed elevated Hcy levels in both the Vit B12-SCD and N_2_O-SCD groups, with no significant difference between them. This finding suggests that while both conditions impair neurological function through disrupted methionine synthase activity, Hcy alone may not be specific enough to differentiate the severity or underlying cause of the neuropathy. It is possible that other factors, such as the direct neurotoxic effects of N_2_O or different biochemical markers, are more responsible for the greater severity observed in the N_2_O-SCD group. Grzych et al. (23) found a correlation between elevated Hcy and axonal damage, which is associated with a poor prognosis, highlighting the direct toxic effect of high Hcy on nerves. Thus, the elevated Hcy levels in N_2_O abusers likely reflect a complex interplay between N_2_O exposure, disrupted vitamin B12 metabolism, and neural damage.

## 5 Conclusion

This study reveals that N_2_O-SCD patients experience more severe neurological damage compared to Vit B12-SCD patients. N_2_O abuse is associated with a higher incidence of psychiatric symptoms and more extensive nerve and spinal cord damage, particularly affecting the lower limbs. While both conditions involve vitamin B12 deficiency, N_2_O not only causes deficiency but also impairs vitamin B12 activity, indicating different pathophysiological and clinical management needs.

## Limitations

This study has several limitations. The sample size in this study was relatively small and the lack of long-term follow-up data further limits the ability to assess disease progression and recovery. Future studies with larger, prospective cohorts are needed to confirm these findings and provide a more comprehensive understanding of the clinical differences between these two forms of SCD. Future studies with larger cohorts and prospective designs are needed to validate our findings and provide further insights into the clinical differences between these two etiologies of SCD.

## Data Availability

The original contributions presented in the study are included in the article/supplementary material. Further inquiries can be directed to the corresponding author.
